# Survey of Demosaicking Methods for Polarization Filter Array Images

**DOI:** 10.3390/s18113688

**Published:** 2018-10-30

**Authors:** Sofiane Mihoubi, Pierre-Jean Lapray, Laurent Bigué

**Affiliations:** 1 Centre de Recherche en Informatique, Signal et Automatique de Lille, Université de Lille—CRIStAL, UMR 9189, 59655 Villeneuve d’Ascq CEDEX, France; sofiane.mihoubi@univ-lille.fr; 2 Institut de Recherche en Informatique, Mathématiques, Automatique et Signal, Université de Haute-Alsace—IRIMAS, EA 7499, 68093 Mulhouse CEDEX, France; laurent.bigue@uha.fr

**Keywords:** polarization filter array, micro-polarizer filter array, spatial interpolation, demosaicking, demosaicing, polarization imaging, division-of-focal-plane polarimeter

## Abstract

Snapshot polarization imaging has gained interest in the last few decades. Recent research and technology achievements defined the polarization Filter Array (PFA). It is dedicated to division-of-focal plane polarimeters, which permits to analyze the direction of light electric field oscillation. Its filters form a mosaicked pattern, in which each pixel only senses a fraction of the total polarization states, so the other missing polarization states have to be interpolated. As for Color or Spectral Filter Arrays (CFA or SFA), several dedicated demosaicking methods exist in the PFA literature. Such methods are mainly based on spatial correlation disregarding inter-channel correlation. We show that polarization channels are strongly correlated in images. We therefore propose to extend some demosaicking methods from CFA/SFA to PFA, and compare them with those that are PFA-oriented. Objective and subjective analysis show that the pseudo panchromatic image difference method provides the best results and can be used as benchmark for PFA demosaicking.

## 1. Introduction

Polarization imaging is a way to analyze the particular direction of oscillation of the electric field described by the light. In opposition with conventional color or multispectral imaging that sample the spectral information, polarization imaging considers the electric field as a vector. Such a vector field is contained in a plane perpendicular to the direction of propagation. As the wave travels, it can oscillate in one particular direction (linear polarization), or in an ellipse (elliptic or circular polarization). Values of polarization images depend on the polarization properties of both the light source and the objects that compose the observed scene. The light can be partially polarized or unpolarized, resulting from either a rapidly changing state of polarization, or an interference effect of polarization.

Several polarization imaging systems, called polarimeters, have been developed in the last past few decades for recovering the polarization state of a lighted scene from few acquisitions. Such systems combine a standard panchromatic imaging device with polarizing optics, e.g., polarization filter, liquid crystal modulator, or prism. Reviews of recent polarimeters have been achieved in the literature [1,2]. The most simple optical setup consists in the rotation of a linear polarization filter at several polarization angles in front of a camera. After a preliminary calibration step (radiometric and polarimetric), the polarization states of the incoming irradiance that reaches the sensor can be estimated. However, this setup is sequential and slow, since several image acquisitions at different filter orientations are required to recover the polarization states of a single scene. To overcome this limitation, Polarization Filter Array (PFA) imaging provides a way for snapshot acquisition that could be useful for many imaging applications. It is an extension of the so-called Color Filter Array (CFA) and Spectral Filter Array (SFA) technologies that previously came on the market. We will briefly review the CFA and SFA technologies and concepts, before to introduce the specificities of PFA.

The CFA technology [3] has quickly become the standard for one-shot color imaging. The technology is lightweight, cheap, robust, and small enough to be embedded in imaging systems. It is composed by a single silicon sensor fitted with a CFA, so that each sensor site senses only one spectral band according to the CFA. A demosaicking procedure is therefore required to recover the incomplete color samples per site. Such procedure uses reflectance properties of acquired images in order to recover the missing color components at each pixel position. Properties of reflectance consist in high spatial correlation in homogeneous areas that constitute an object, and spectral correlation between different channels. The widely-used Bayer CFA for instance samples the green band at half of sites, which makes it a prominent candidate to begin the demosaicking process using spatial correlation. Spectral correlation is then generally assumed in order to estimate red and blue channels using the well estimated green channel. The demosaicking algorithm has to be carefully selected since color reconstruction quality is highly affected by its artifacts, such as blur, zipper effect, etc.

The last past few decades have seen the emergence of an extension of CFA with more than three channels: The SFA technology [4,5,6]. Supplementary channels are generally required for applications that need good color reproduction [7], illuminant estimation and spectral adaptation [8], reflectance reconstruction [9], etc. SFA design considers a trade-off between spatial resolution for spatial reconstruction in the demosaicking process, and spectral resolution for spectrum reconstruction. Thus, some SFA demosaicking algorithms privilege spatial resolution by sampling a dominant channel that represents half of pixels [10] (as for the Bayer CFA), while other privileges spectrum reconstruction by maximizing the number of channels [11].

Polarization Filter Array (PFA) technology has been patented in 1995 [12], but most of the practical implementations and technology advances were made from 2009 to nowadays. Manufacturing processes are various and done by designing metal wire grid micro-structures [13,14,15], liquid crystals [16,17], waveplate array of silica glass [18], or intrinsically polarization-sensitive detectors [19,20]. Some of the most evolved PFA structures are presented as being bio-inspired, and implement additional features, e.g., mixing spectral and polarization feature recovery [21], high dynamic range [22], or motion detection [23].

**Figure 1:**
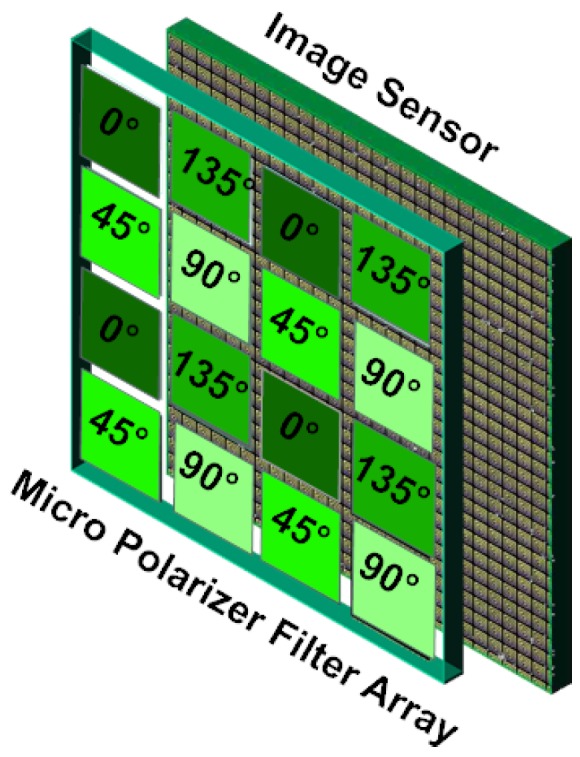
Polarization Filter Array principle. A polarization filter array covers the pixel matrix of a radiometric sensor. The array of polarimetric filters are located, either directly above the matrix of pixels, or over the micro-lens array.

The PFA is composed of pixel-size linear polarizers oriented at four different angles ($0^{\circ }$, $45^{\circ }$, $90^{\circ }$, and $135^{\circ }$ are the polarization orientations employed in most of the PFA cameras), superimposed on a camera sensor chip, as shown in Figure 1. In front of the sensor, the PFA samples the polarization direction by filtering the incoming irradiance according to polarizer angles. Therefore, each pixel measures the intensity coming from only 1 of the 4 different polarizers. Some PFA cameras appear on the market, like the Polarcam device from 4D Technology [24], and more recently, the IMX250MZR polarization-dedicated sensor from SONY (Tokyo, Japan). Both PFA use the same filter arrangement that is described in Figure 1. But the SONY sensor that comes in 2018 is particularly cheap, and holds the polarization matrix bellow the lens array, which limits the cross-talk effect in adjacent pixels [6]. Moreover, as it was previously done for other computational imaging [25,26] and computer vision [27] algorithms, Lapray et al. [2] have recently proposed an implementation of a real-time polarization imaging pipeline using an FPGA.

Demosaicking PFA images aims to retrieve the full resolution images that represent the four polarization channels. Stokes imaging is a tool that uses these channels to represent in an efficient way the linear and circular state of polarization of the incoming light. Thus, the final goal of demosaicking is to minimize errors and artifacts in the reconstructed Stokes parameters and the derived descriptors. The Degree Of Linear Polarization (DOLP) and the Angle Of Linear Polarization (AOLP) descriptors are computed from the first three Stokes parameters of the Stokes vector S. In this work, we limit ourself to the linear case, as the most of existing PFA are based only on linear polarizers (but some existing tentatives add plasmonic quarter-wave retarders to retrieve the circular polarization component [28]). Let us consider the intensities of light measured $I^{0}$, $I^{45}$, $I^{90}$, $I^{135}$ after the light is filtered by linear polarization filters (oriented at 0°, 45°, 90°, and 135°). In the literature, the choice of these 4 angles forms an optimal system for polarization imaging in the presence of noise, as described in [29]. The mathematical formulations for Stokes parameters and descriptors are as follows:

(1) $$S=\left[\begin{array}{c} S_{0}\\ S_{1}\\ S_{2}\\ 0 \end{array}\right]=\left[\begin{array}{c} I^{0}+I^{90}\\ I^{0}-I^{90}\\ I^{45}-I^{135}\\ 0 \end{array}\right],$$

(2) $$DOLP=\frac{\sqrt{S_{1}^{2}+S_{2}^{2}}}{S_{0}},$$

(3) $$AOLP=\frac{1}{2}\arctan\frac{S_{2}}{S_{1}},$$

The total incoming irradiance is represented by $S_{0}$, $S_{1}$ is the horizontal/vertical polarization difference, whereas $S_{2}$ is the +45/−45° polarization difference. If we consider that channels $I^{k}$, k∈{0,45,90,135} are normalized intensity values comprised between 0 and 1, $S_{1}$ and $S_{2}$ have values between −1 and +1. AOLP values are scaled in the range $\left[0,180^{\circ }\right]$, whereas DOLP values are scaled in the range [0,1], and are often expressed in percentage of polarized light.

**Figure 2:**
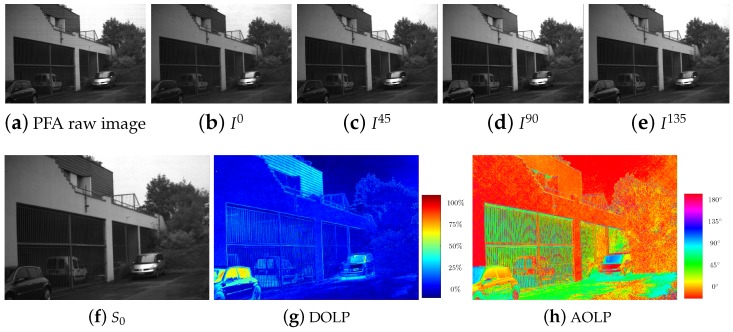
Polarization Filter Array (PFA) imaging overview. (**a**) Raw output image from the 4D Technology camera. (**b**–**e**) Downscaled polarization images (without spatial interpolation). (**f**–**h**) Polarization descriptor images associated to downsampled images.

It is useful to note that a radiometric calibration is very important in case of polarimetric imaging, even more than for color imaging, as the different channel errors are coupled, and thus it can invalidate greatly the parameter estimation [1]. An example of a complete 2D Stokes processing starting from a PFA image is given in Figure 2.

**Figure 3:**
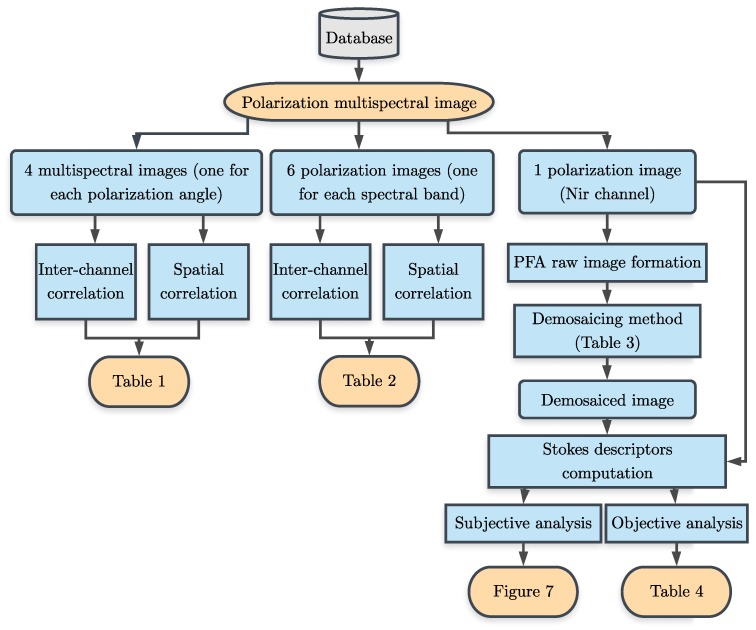
Overview of the analysis which is done in this paper.

The purpose of this paper is to first study the correlation properties of polarization channels and their similarities with those of spectral channels. Then to review some existing interpolation strategies dedicated to filter array imaging, i.e., CFA, SFA, and PFA. Finally, we propose to evaluate objectively the methods and those we have adapted to the PFA case, in the special context of PFA. A diagram of the proposed analysis is shown in Figure 3. We organize the paper as follows. First, a data correlation study across the polarization channels is presented in Section 2. Next, different CFA, SFA, and PFA interpolation techniques are presented in Section 3. Results and discussion of the surveyed methods is proposed in Section 4. The paper ends with several conclusions in Section 5.

## 2. Polarimetric Channel Correlation Study

All demosaicking methods estimate missing values using spatial (intra-channel) (i) and/or inter-channel (ii) correlation assumptions. (i) The spatial correlation assumes that; if a pixel *p* and its neighborhood belong to the same homogeneous area, the value of *p* is strongly correlated with the values in its neighborhood. Thus, assuming that a channel is composed of homogeneous areas separated by edges, the value of a pixel can be estimated by using its neighbors within the same homogeneous area. Spatial gradients are often used as indicators to determine whether two pixels belong to the same homogeneous area. Indeed, gradient considers the difference between values of two spatially close pixels. We can therefore assume that these pixels belong to the same homogeneous area if the gradient is low, and that they belong to different homogeneous area otherwise. (ii) The inter-channel correlation (also called spectral correlation in CFA and SFA imaging) assumes that the high frequencies (textures or edges) of the different channels are strongly correlated. If the filter array contains a spatially dominant band, demosaicking generally estimates the associated channel whose high frequencies can be faithfully reconstructed, then uses it as a guide to estimate other channels. The faithfully reconstructed image can be used to guide the high frequency estimation within the different channels [30].

Usual PFA demosaicking methods assume only spatial correlation, thus disregarding correlation among polarization channels. In order to extend CFA and SFA demosaicking methods that also use the inter-channel correlation to PFA images, we propose to compare the spatial and inter-channel correlations in multispectral images with those of polarization images. For this purpose, we use the database proposed in [31]. Images were acquired by the single-band near-infrared sensor from the JAI AD080 GE camera, coupled with a linear polarizer from Thorlabs (LPNIRE100-B). A precision motorized rotation stages (Agilis™ Piezo Motor Driven Rotation Stages) allowed to take the four images at four orientation angles (${\left[I^{0},I^{45},I^{90},I^{135}\right]}^{T}$). A registration procedure aligned the images [32] pixel-to-pixel. The images were also calibrated with respect to the spatial deviation of the illuminant and the non-linearities. There are ten multispectral images, each one being provided with four different polarization angles k∈{0,45,90,135}. Scenes imply different objects with materials like fabrics, plastics, food, color checkers, glass, and metal. Conditions of acquisition are constant for all scenes, i.e., constant illuminant source (tungsten halogen source) and location, constant field of view and constant lens aperture. Multispectral recoverred images are composed of six spectral channels: Five channels are associated with the visible domain, whereas one channel is associated with the Near-InfraRed domain (NIR). The six spectral channels $C^{u}$ are arranged so that their associated spectral band wavelengths increase with respect to u∈{1,…,6}.

Let us first study the properties of multispectral images with respect to the polarization angle of analysis. For this purpose we assess the spatial correlation within a given channel $C^{u}$ using the Pearson correlation coefficient (PCC) between the value $C_{p}^{u}$ of each pixel *p* and that of its right neighbor $C_{q}^{u}$ at spatial distance 2. This coefficient is defined as [33]

(4) $$PCC\left[C^{u}\right]=\frac{\sum_{p}\left(\left(C_{p}^{u}-\mu^{u}\right)\left(C_{q}^{u}-\mu^{u}\right)\right)}{\sqrt{\sum_{p}{\left(C_{p}^{u}-\mu^{u}\right)}^{2}}\sqrt{\sum_{p}{\left(C_{q}^{u}-\mu^{u}\right)}^{2}}},$$

where $\mu^{u}$ is the mean value of channel $C^{u}$. We also assess the inter-channel correlation using the PCC between any pair of spectral channels $C^{u}$ and $C^{v}$, $\left(u,v\right)\in {\left\{1,\ldots ,6\right\}}^{2}$ as

(5) $$PCC\left[C^{u},C^{v}\right]=\frac{\sum_{p}\left(\left(C_{p}^{u}-\mu^{u}\right)\left(C_{p}^{v}-\mu^{v}\right)\right)}{\sqrt{\sum_{p}{\left(C_{p}^{u}-\mu^{u}\right)}^{2}}\sqrt{\sum_{p}{\left(C_{p}^{v}-\mu^{v}\right)}^{2}}}.$$

Note that in Equations (4) and (5), we select a centered area excluding the 16 pixels on the image borders to avoid border effects, that are induced by the registration step used on raw images (described in [31]). Moreover the choice of 16 border pixels is done to match with the experimental assessment (see Section 4) of demosaicking methods presented in Section 3.

Table 1 is the spatial correlation within each spectral channel and the inter-channel correlation between the six spectral channels according to each of the four polarization angles. Table 1 shows that the spatial correlation is relatively high (0.9504 on average over all channels and polarization angles), which validates the use of the spatial correlation assumption for both SFA and PFA demosaicking. According to Table 1a,d, the spatial correlation has the same behavior for the four polarization angles. It also highlights that the channel $C^{4}$ has low spatial correlation. We believe that it is due to the database acquisition setup, which uses the dual-RGB method leading to unbalanced channel sensitivities. In this configuration, the spectral sensitivity function associated with the channel $C^{4}$ is lower than other channels over the spectrum. Thus, all channels don’t share the same noise level, and poor information recovery (especially for $C^{4}$) could lead to low correlation values.

Regarding the spectral inter-channel correlation, the usual behavior is that close spectral channels in term of wavelength band are more correlated than distant ones, and channels in the visible are weakly correlated with the near-infrared channel [11]. Except the channel $C^{4}$ that exhibit low correlation values, this behavior is observed in Table 1. Indeed, $PCC\left(C^{1},C^{2}\right)>PCC\left(C^{1},C^{3}\right)>PCC\left(C^{1},C^{5}\right)>PCC\left(C^{1},C^{6}\right)$ for instance. Moreover the correlation between the NIR channel $C^{6}$ and other channels is low (ranges on average between 0.7953 and 0.8787), while the correlation between channels in the visible domain reaches up to 0.9596 (correlation between $C^{2}$ and $C^{3}$). Table 1a,d show that the inter-channel correlation depends on the polarization angle. Indeed, Table 1a has values close to Table 1d, whereas Table 1b has values close to Table 1c. We can therefore expect that the polarization channels at 0° are more correlated with those at 135° than those at 45° or 90°.

Now, let us consider the polarization images composed of four polarization angles for a given spectral band. The spatial and inter-channel correlations are assessed using the *PCC* applied respectively to channels $I^{k}$, k∈{0,45,90,135} (see Equation (4)), and to any pair of polarization channels $I^{k}$ and $I^{l}$, $\left(k,l\right)\in {\left\{0,45,90,135\right\}}^{2}$ (see Equation (5)).

Table 2 is the average polarization correlation between the four channels of polarization images, according to each of the six spectral bands. Results highlight that the spatial correlation is high and does not depend on the considered spectral band (except for channel $C^{4}$). Results also confirm that channel $I^{0}$ is highly correlated with channel $I^{135}$ and channel $I^{45}$ is highly correlated with channel $I^{90}$. In general terms, inter-channel correlation between polarization channels is higher than inter-channel correlation between spectral channels (see Table 1). Indeed, if the incoming irradiance is not polarized, the associated pixel has only the information of the total intensity divided by two, that is the same from one channel to another.

Since the inter-channel correlation is high in polarization images, we propose to apply SFA demosaicking schemes based on inter-channel correlation assumption on PFA images. For this purpose, we can choose the four polarization channels associated to any spectral band but not the one associated to $C^{4}$. Since dual-RGB method is not applied for the channel $C^{6}$, we selected it for the experimental assessment in Section 4.

## 3. State-of-the-Art

### 3.1. Demosaicking Problem and Properties

A PFA camera provides a raw image $I^{raw}$ with X×Y pixels, in which a single polarization angle k∈{0,45,90,135} is available at each pixel *p* according to the PFA arrangement. Let *S* be the set of all pixels (with a cardinal of |S|=X×Y) and $S^{k}$ be the pixel subset where the PFA samples the angle *k*, such that $S={⋃}_{k\in \{0,45,90,135\}}S^{k}$. A PFA can be defined as a function PFA:S→{0,45,90,135} that associates to each pixel *p* its polarization angle. Therefore the pixel subset where the PFA samples the polarization angle *k* can be defined as $S^{k}=\left\{p\in S,PFA(p)=k\right\}$. The raw image $I^{raw}$ can then be seen as a sampled version of the fully-defined reference image $I={\left\{I^{k}\right\}}_{k\in \{0,45,90,135\}}$ (that is unavailable in practice) according to the PFA:

(6) $$\forall p\in S,\;I_{p}^{raw}=I_{p}^{PFA(p)}.$$

The raw image can also be seen as the direct sum of four sparse channels ${\tilde{I}}^{k}$, k∈{0,45,90,135} that contains the available values at pixel positions in $S^{k}$ and zero elsewhere:

(7) $${\tilde{I}}^{k}=I^{raw}⊙m^{k},$$

where ⊙ denotes the element-wise product and $m^{k}$ is a binary mask defined at each pixel *p* as:

(8) $$m_{p}^{k}=\left\{\begin{array}{cc} 1 & \mathrm{if}\;PFA\left(p\right)=k,\;i.e.,\;p\in S^{k},\\ 0 & \mathrm{otherwise}. \end{array}\right.$$

**Figure 4:**
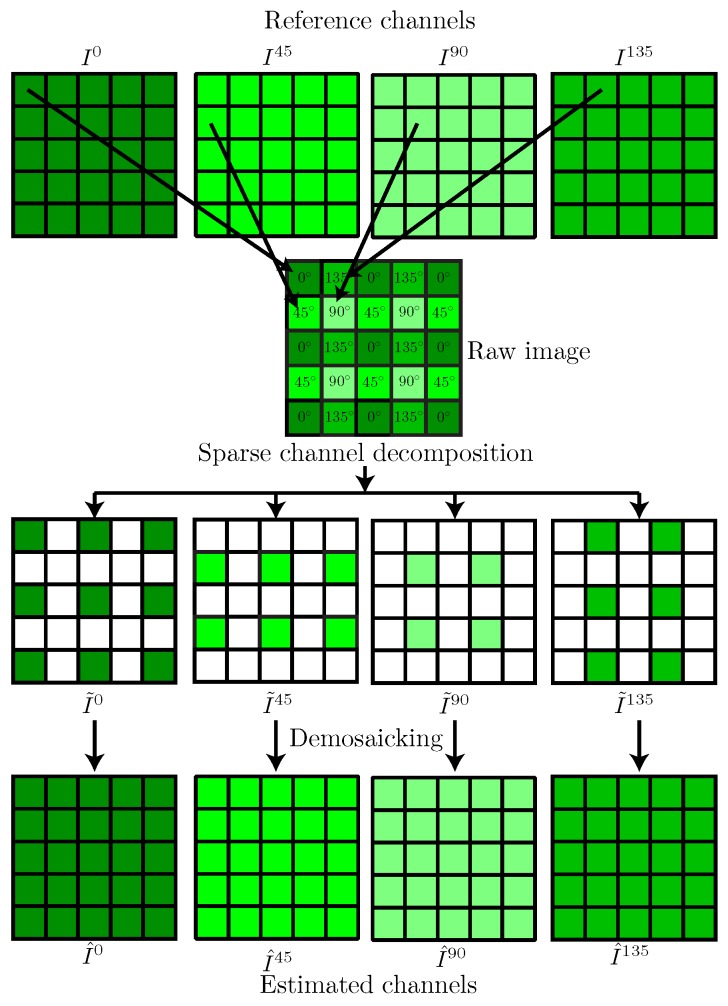
General mosaicking/demosaicking testing framework used in this work.

Demosaicking is performed on each sparse channel ${\tilde{I}}^{k}$ to obtain an estimated image $\hat{I}={\left\{{\hat{I}}^{k}\right\}}_{k=1}^{K}$ with four fully-defined channels, among which three are estimated at each pixel *p*. For illustration purpose, Figure 4 shows the demosaicking problem formulation for a PFA raw image.

In the following, we review the demosaicking methods dedicated to PFA. We also review those dedicated to CFA/SFA that can be used or adapted to our considered PFA. All these methods were either re-coded, adapted to PFA, or kindly provided by authors (online or in private). See Table 3 for an overview of all methods.

### 3.2. PFA Demosaicking

Among PFA demosaicking methods, we exclude the learning-based methods [46], since they require well-adapted dictionaries, and methods that exploit multiple sampling of the raw data [47]. We also exclude the gradient-based method [48], since a SFA method has a very close behavior (BTES).

#### 3.2.1. Bilinear with 5 Different Kernels (B)

**Figure 5:**
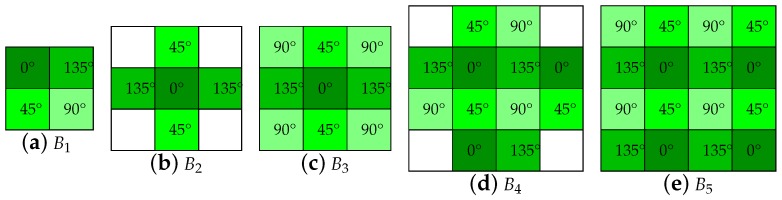
The five demosaicking kernels of the five bilinear methods $B_{1-5}$ from the work by Ratliff et al. [34]. It refers to the neighborhood used for interpolation. (**a**–**c**) Are simple bilinear kernels, whereas (**d**,**e**) are weighted bilinear kernels.

Bilinear interpolation dedicated to PFA was firstly investigated by Ratliff et al. [34]. They employ three different bilinear and two weighted bilinear kernels (see Figure 5). Bilinear interpolation is one of the most commonly used technique due to its low computational complexity. It is based on space-invariant linear filtering. Two kinds of bilinear interpolations exist. One uses a linear combination of neighboring pixel values using equal weights. Another employs non-equal weights in accordance to the Euclidean distance between the interpolated pixel location and centers of neighboring pixels. The subtractive nature of the Stokes vector processing results in strong edge artifacts in the reconstructed images. Based on this assumption, authors define the term of Instantaneous Field-Of-View (IFOV), which is the local deviation between an ideal full-resolution polarimeter and the interpolated PFA pixel responses at each position. They evaluate the interpolation performance of the methods using synthetic data, in the frequency domain of the reconstructed Stokes images. As evaluation metrics, they computed the modulation and intermodulation transfer functions in the descriptor images, along with the DOLP Mean Squared Error (MSE). It is found that the larger the size of the kernels becomes, the more DOLP artifacts are reduced, at the cost of loosing the high spatial frequency features. They found that the 12-pixel neighborhood kernel ($B_{4}$ in the Figure 5) gives the best performance in term of IFOV removal. For algorithm implementations, we used the same weights as the original paper for the two weighted bilinear kernels.

#### 3.2.2. Linear System (LS)

Tyo et al. [35], in 2009, elaborates a new method to reconstruct the first three Stokes parameters directly from the mosaicked image, without estimating $\hat{I}$. The four polarization images ${\hat{I}}^{0}$, ${\hat{I}}^{45}$, ${\hat{I}}^{90}$, and ${\hat{I}}^{135}$ are thus not available with this method. The philosophy starts from the analysis of a raw PFA image in the frequency domain. By doing the discrete 2D Fourier transform, they define the spatial low-pass and high-pass filters. They assume that $S_{0}$, $S_{1}$, and $S_{2}$ are spatially band limited in the frequency domain. The centering baseband of the Fourier transform represents $S_{0}$, whereas the horizontal and vertical sidebands represent $S_{1}+S_{2}$ and $S_{1}-S_{2}$ respectively. They could then reconstruct $S_{1}$ and $S_{2}$ after applying the filters in the Fourier domain, and by doing the inverse Fourier transform of images.

#### 3.2.3. Adaptive (A)

An extension of the bilinear interpolation was proposed by Ratliff et al. [36]. In this work, the principle is inspired by Ramanath et al. [49]. The loss of high frequency features in bilinear interpolation techniques is compensated by local computation using a 3×3 filtering. First, $S_{0}$ is approximated using not only $I_{0}$ and $I_{90}$, but the four available neighboring intensities, as it is suggested in the literature [50]. A 2×2 low-pass filtering of the raw PFA image is performed with the kernel as follows:

(9) $$h_{S_{0}}=\frac{1}{2}\cdot \left[\begin{array}{cc} 1 & 1\\ 1 & 1 \end{array}\right].$$

Then, intensity similarity masks and Euclidian distance masks are computed, in such a way that the weights are higher for pixels that have similar intensity values within a close neighborhood. These local interpolation weights are computed at each position in the image, and avoid interpolation across edges, and thus preserve high frequency contents. Results show that IFOV artifacts and false edges are minimized in the DOLP image, while high spatial frequencies are preserved. Only a subjective evaluation of their algorithm is performed in the article. The parameter $\rho_{i}$ in the paper was selected to be equal to 0.3 in our implementation.

#### 3.2.4. CuBic (CB) and Cubic-SPline (CBSP)

An article was published by Gao et al. [37] to compare bilinear, weighted bilinear, cubic, and cubic-spline. In our work, the cubic and bicubic interpolation methods have been implemented using built-in functions from Matlab software (The MathWorks, Inc., Natick, MA, USA). Cubic interpolation uses the third order polynomial fitting to interpolate an area delimited by four corners, and uses three directional derivatives (horizontally, vertically and diagonally) as input. The cubic-spline method is a sequence of an horizontal interpolation and a vertical interpolation. Polynomial fitting (third order) is also used to reconstruct missing values from adjacent pixels, but with the additional constraint that the first and second derivative at the interpolation points are continuous. A modulation transfer function study is done to investigate on how the high spatial frequencies are preserved. A visual and objective evaluation (using MSE) are done on real data. Main results show that the cubic-spline methods performed the best in terms of visual artifacts removal and MSE. It appears that bilinear and weighted bilinear give the worst results.

#### 3.2.5. Intensity Correlation among Polarization Channels (ICPC)

Another method by Zhang et al. [38] takes advantage of the correlations in PFA to enhance the spatial resolution of images. Spatial and polarization correlations between channels are investigated in a close pixel neighborhood, directly in the raw PFA image. Edges can not be accurately distinguished if the incoming light is polarized at some degree. Thus, in their work, edge orientations are estimated using the intensity correlation. They start by computing correlation errors from the assumption that edges have poor correlation within the pixel neighborhood. The correlation error magnitude reflects the presence of a homogeneous zone, or of a horizontal, vertical, or diagonal edge. For the interpolation in itself, a diagonal interpolation is firstly done by applying a bicubic-spline interpolation. Then, horizontal and vertical interpolation are performed by bicubic-spline interpolations, according to the correlation errors previously computed. Evaluation of their method is done by constructing a set of four ground truth polarization images using a linear polarizer rotated at four different angles. They found that their method performs better visual results, and has better RMSE compared to bilinear, bicubic, bicubic-spline, and gradient-based methods.

### 3.3. CFA Demosaicking

Bayer CFA has a dominant green band that represents half of pixels, and is used as a guide containing the high spatial frequencies. Therefore, CFA demosaicking methods generally estimate the green channel first in order to use the spectral correlation by considering that green channel is strongly correlated with blue and red channels. Here, we extend residual interpolation methods [39,40] from the CFA to the PFA pattern by considering the intensity image $S_{0}$ as a guide instead of the estimated green channel.

#### 3.3.1. Residual Interpolation (RI)

Kiku et al. [39] propose a demosaicking scheme based on the residual interpolation. Their method requires a well estimated guide image, i.e., the estimated green channel that is dominant in the Bayer CFA raw image. Since there is no dominant band in our considered PFA, we adapt their method by using the intensity image $S_{0}$ as a guide. It is well estimated from a simple 2×2 bilinear kernel (see Equation (9)). Each channel ${\hat{I}}^{k}$ is then recovered by following these successive steps:

1. It computes a tentative estimated channel ${\overset{ˇ}{I}}^{k}$ by applying the guided filter [51] and the guide image to the sparse channel ${\tilde{I}}^{k}$. Note that such process modifies the raw values in the tentative estimated channel ${\overset{ˇ}{I}}^{k}$.
2. It computes the residuals defined by a difference between ${\tilde{I}}^{k}$ and tentatively estimated channel ${\overset{ˇ}{I}}^{k}$ at pixels in $S^{k}$.
3. It performs a bilinear interpolation of the residuals by using $B_{3}$ filter.
4. The finally estimated channel ${\hat{I}}^{k}$ is given by the summation of the tentative estimated channel ${\overset{ˇ}{I}}^{k}$ and the interpolated residuals.

#### 3.3.2. Adaptive Residual Interpolation (ARI)

Monno et al. [40] improve the RI by applying a Laplacian filter on ${\tilde{I}}^{k}$ and the guide before using the guided filter. The parameters for RI and ARI implementations are h=5, v=5, and ϵ=0.

### 3.4. Spectral Demosaicking Methods for a 2×2 Pattern

Among SFA demosaicking methods, we exclude learning-based methods since they require fully-defined images [30,52,53], and methods that assume sparsity of the raw data [54,55,56].

#### 3.4.1. Binary-Three Edge Sensing (BTES)

**Figure 6:**
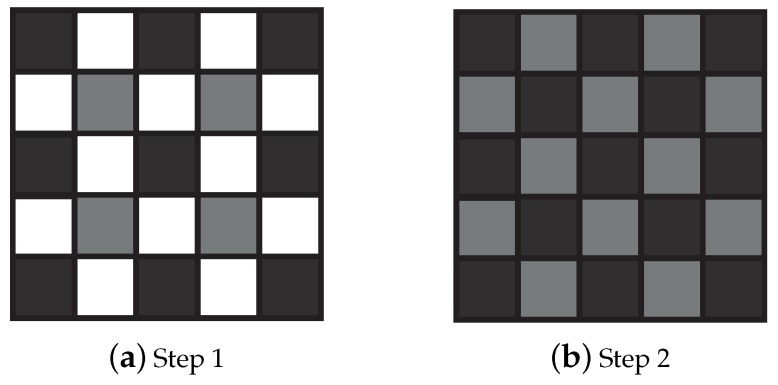
Neighborhood used for weight computation in any channel according to the step of binary-three edge sensing (BTES) algorithm. Pixels in black are known or previously estimated, whereas pixels in gray are the estimated pixels. Pixels in white are unknown and not estimated at the current step.

In our knowledge, BTES interpolation [41] is the first SFA demosaicking method applicable on PFA raw images. This method improves the bilinear interpolation by considering weights inversely proportional to the directional gradient. It follows two steps, in which only a subset of pixels are estimated as shown in Figure 6. In a first step, a quarter of pixels are estimated using their four closest neighbors weighted with respect to the diagonal gradients. In a second step, the rest of the pixels ($\frac{card(S)}{2}$) are estimated using their four closest neighbors weighted with respect to horizontal (for an horizontal neighbor) or vertical (for a vertical neighbor) gradients.

As bilinear interpolation, this method is only based on spatial correlation since there is no dominant channel. Other SFA demosaicking methods also consider the inter-channel correlation to estimate the missing channels.

#### 3.4.2. Spectral Difference (SD)

Brauers and Aach [42] estimate missing values of a channel using the inter-channel correlation. They consider the available value in the raw image at the missing position, i.e., a pixel *p* of a channel $I^{k}$ is estimated using the information of channel $I^{PFA(p)}$ as follows:

1. It computes the sparse difference channel ${\tilde{\Delta }}^{k,PFA(p)}$ between channel $I^{k}$ and the channel ${\hat{I}}_{B_{3}}^{PFA(p)}$ estimated by bilinear interpolation (using filter $B_{3}$) at pixels in $S^{k}$.
2. It estimates the fully-defined difference channel ${\hat{\Delta }}_{B_{3}}^{k,PFA(p)}$ by bilinear interpolation.
3. The value of ${\hat{I}}_{p}^{k}$ is given by the sum between the difference channel ${\hat{\Delta }}_{B_{3}}^{k,PFA(p)}$ and the available value at *p* in the raw image.

Mizutani et al. [57] further improve this method using an additional assumption: Spectrally close channels are more correlated than distant ones. Since this assumption is not validated for polarization images, we cannot use it in this context.

#### 3.4.3. Vector Median (VM)

Wang et al. [43] consider that each pixel of an image as a vector with four dimensions. For each pixel *p*, the method defines many pseudo-pixels by column vectors (${\left[I_{p}^{0},I_{p}^{45},I_{p}^{90},I_{p}^{135}\right]}^{T}$ in our case) according to the mosaic, and it affects the median pseudo-pixel to *p*. The pseudo-pixels at *p* represents all the possible combinations of the four channels in a 5×5 neighborhood around *p*. The four values of a pseudo-pixel are taken from spatially connected pixels. To preserve value discontinuities and color artifacts, authors also propose a post-processing in a 3D-spheric space.

#### 3.4.4. Discrete Wavelet Transform (DWT)

Wang et al. [44] extend the DWT-based CFA demosaicking to SFA demosaicking. By considering an image as low-frequency (homogeneous areas) and high-frequency contents (edges), This approach assumes two things: The low-frequency content is well estimated by bilinear interpolation, and the high-frequency contents have to be determined more accurately and have to be the same in different channels. The algorithm first estimates a fully-defined multispectral image ${\hat{I}}_{B_{3}}$ by bilinear interpolation, then applies five successive steps to each channel ${\hat{I}}_{B_{3}}^{k}$ as follows:

1. It decomposes ${\hat{I}}_{B_{3}}^{k}$ into *K* Down-Sampled (DS) images, so that each DS image is composed of pixels in $S^{l}$, l∈{0,45,90,135}. Note that one DS image is only composed of raw values.
2. It decomposes each DS image into spatial frequency sub-bands by DWT using Haar wavelet D2.
3. It replaces the spatial high-frequency sub-bands of all estimated DS images by those of the corresponding DS images of the mid-spectrum channel, assuming this is the sharpest one. In PFA images, there is no mid-spectrum channel, we therefore propose to use arbitrarily the ${\hat{I}}_{b_{3}}^{90}$ channel.
4. DS images are transformed by inverse DWT.
5. It recomposes the full-resolution channel ${\hat{I}}^{k}$ from the four transformed DS images.

#### 3.4.5. Multi-Local Directional Interpolation (MLDI)

Shinoda et al. [45] combine BTES and SD approaches into the MLDI method that follows the two steps of BTES. Each pixel is estimated using the difference planes, as in SD scheme. Moreover, instead of simply estimating the fully-defined difference planes by bilinear interpolation, the authors use directional gradients (following the step in BTES scheme), which improves the estimation. Shinoda et al. [45] also propose a post-processing that updates each estimated channel by taking into account the previous estimated values.

#### 3.4.6. Pseudo-Panchromatic Image Difference (PPID)

The Pseudo-Panchromatic Image (PPI) is defined in each pixel as the average of all channels. By assuming that PPI values of neighboring pixels are strongly correlated, Mihoubi et al. [11] estimate the PPI from the PFA image by applying an averaging filter *M* proposed in [58]. Such filter estimates the PPI as the average value of all channels in a given neighborhood of each pixel. For this purpose, it takes all channels into account, while being as small as possible to avoid estimation errors. For our considered PFA arrangement, the filter *M* is adapted as:

(10) $$M=\frac{1}{16}\cdot \left[\begin{array}{ccc} 1 & 2 & 1\\ 2 & 4 & 2\\ 1 & 2 & 1 \end{array}\right].$$

In the case of strong spectral correlation (≥0.9), authors propose to restore the edges of the estimated PPI using directional gradients. However, the condition is not validated for PFA images. The estimated PPI is thereafter used in a PPI difference scheme that estimates each channel *k* as follows:

1. It computes the sparse difference channel ${\tilde{\Delta }}^{k,PPI}$ between channel $I^{k}$ and the PPI at pixels in $S^{k}$.
2. It estimates the fully-defined difference channel ${\hat{\Delta }}^{k,PPI}$ by weighted bilinear interpolation in which the weights are inversely proportional to the gradients computed from the raw image.
3. The finally estimated channel ${\hat{I}}^{k}$ is the sum between the estimated PPI and the difference plane.

#### 3.4.7. Pseudo-Panchromatic Image based Discrete Wavelet Transform (PPIDWT)

The PPI has similar information to the mid-spectrum channel, and it is better estimated. Mihoubi et al. [11] therefore propose to replace the spatial high-frequency sub-bands by those of the PPI instead of $I_{b_{3}}^{90}$ channel in the DWT scheme.

## 4. Performance Evaluation of Demosaicking Algorithms

### 4.1. Experimental Setup

PFA image simulation is employed to assess the interpolation strategies. As for the correlation study in Section 2, the polarimetric images from the database of Lapray et al. [31] was used as references.

All methods of Table 3 were either re-coded (R), adapted to PFA (A), or provided by authors in Matlab/ImageJ language software (P). They are further integrated into the framework presented in Figure 4 in order to assess and compare the performances of demosaicking. Stokes descriptors are then computed for both reference and estimated images, according to Equations (1)–(3). To avoid precision errors during image conversions, all considered images and processing are using 32-bit float data representation.

We consider the Peak Signal-to-Noise Ratio (PSNR) as quality metric. Between each couple of reference (R) and estimated (E) channel/descriptor, the PSNR is computed as follows:

(11) $$PSNR\left(R,E\right)=10\log_{10}\left(\frac{{\left(\max_{p}R\right)}^{2}}{MSE(R,E)}\right),$$

where MSE(R,E) denotes the mean squared error between *R* and *E*. Because $\max_{p}R$ can differ from a channel (or a descriptor) to another, Equation (11) takes into account this actual maximal level rather than the theoretical one to avoid misleading PSNR values. In PSNR computation, as for the previous correlation study, we exclude the 16 pixels in each of the four borders of the image to avoid inherent border effect related to either registration or demosaicking processing.

### 4.2. Results and Discussion

Table 4 displays the PSNR values provided by the demosaicking methods on average over the ten database scenes. Results show that among bilinear filters, $B_{3}$ provides the best results for $I^{0}$, $I^{45}$, $I^{90}$, $I^{135}$, $S_{0}$, $S_{1}$, and DOLP, while $B_{4}$ slightly exceeds it for S2 and AOLP. Among PFA-oriented methods, CB and CBSP generally provide the best results. Our proposition to adapt RI and ARI CFA demosaicking methods to the PFA case (with $S_{0}$ as guide) provides better results than classical PFA-oriented methods. We remark that RI and ARI are very close together in the PSNR results. RI also provides the best results among all tested methods regarding $S_{2}$ parameter and DOLP descriptor.

For PFA methods, it is important to note that the output interpolated pixel is shifted by half pixel when using bilinear kernels $B_{1}$, $B_{4}$, and $B_{5}$, compared to other bilinear kernels $B_{2}$, $B_{3}$. The output pixel is either aligned to the original interpolated pixel position center, or in the pixel boundaries. We did not correct for this misalignment because applying an image translation by half pixel needs an additional cubic or linear interpolation. So such a registration process cannot be used as a pre-processing for an acceptable comparison methodology over the bilinear demosaicking methods. Thus, the results for $B_{1}$, $B_{4}$, and $B_{5}$ should be interpreted with care.

For tested SFA-oriented methods, the use of spectral correlation generally provides better performance than simple bilinear interpolations. Moreover, methods based on gradient computation (BTES, MLDI, and PPID) exhibit the best demosaicking performances. By considering the PPI as a guide for demosaicking, PPID shows the best demosaicking performances among all methods for all polarization channels, also for $S_{0}$, $S_{1}$ parameters and AOLP descriptors.

**Figure 7:**
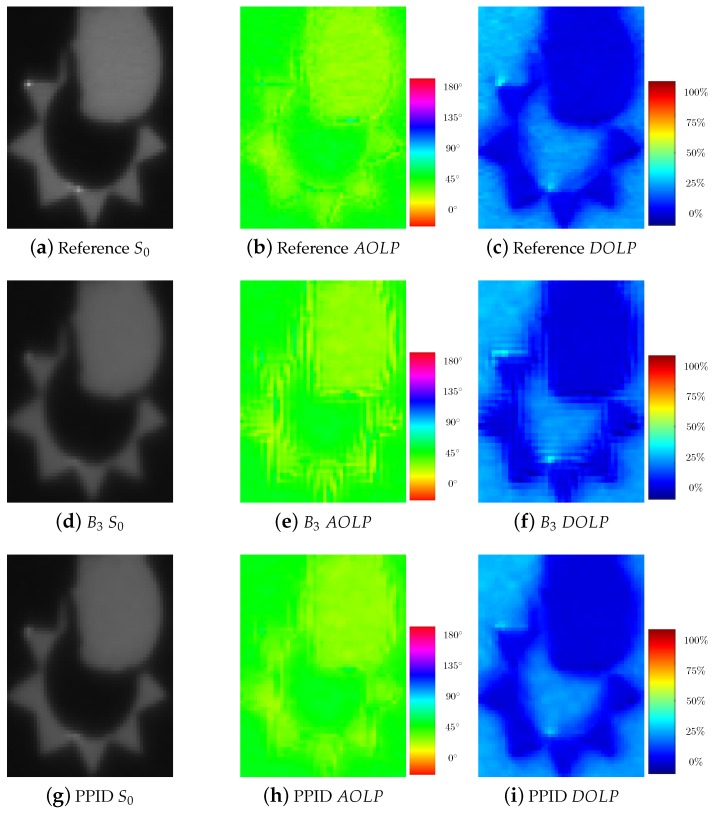
Zoom in of the “macbeth_enhancement” scene from the database of Lapray et al. [31]. (**a**–**c**) Images resulting $S_{0}$, angle of linear polarization (*AOLP*), and degree of linear polarization (*DOLP*) processed using the full-resolution reference. (**d**–**f**) The bilinearely interpolation [34] ($B_{3}$). (**g**–**i**) The pseudo-panchromatic image difference (PPID) interpolation [11].

To visually compare the results provided by demosaicking methods on $S_{0}$, AOLP, and DOLP descriptors, we select a zoomed area from the “macbeth_enhancement” scene of the database. Among demosaicking methods, we show the results of the most intuitive method (bilinear interpolation using $B_{3}$ filter), and the pseudo panchromatic image difference (PPID) that globally provides the best PSNR results. Figure 7 shows that there is no significant difference regarding the $S_{0}$ parameter, except that the two highlight dots are more apparent in PPID demosaicked image. Computing AOLP and DOLP parameter from a bilinearly interpolated image generates many artifacts that are fairly reduced using PPID demosaicking method.

Generally speaking, we found that demosaicking that are dedicated to PFA don’t necessary give better PSNR result. Thus, it was not obvious that considering color and spectral demosaicking techniques applied to PFA arrangement could be beneficial. The results highlights that this can benefit the pre-processing of PFA.

However, we can express some reservations about the results obtained. First, we limited our study on a relatively small database. Other polarization database in the literature [59] furbish only the Stokes parameters and polarization descriptors, but no fully-defined reference image $I={\left\{I^{k}\right\}}_{k\in \{0,45,90,135\}}$ are available. Natural scene samples could also be beneficial for a complementary algorithm classification. Secondly, the database used in this work was made with the same experimental conditions, i.e., constant angle/plan of incidence and a unique non-polarized illuminant. We think that supplementary tests of the best identified algorithms in an extended database containing a better statistical distribution of data could be valuable. Thirdly, the noise associated with reference images is not quantified, and is slightly different from a PFA acquisition system. We thus disregarded recent denoising-demosaicking algorithms that estimate sensor noise to improve the accuracy of demosaicking [60,61,62,63].

The arrangement of the filter array investigated consists in a 2×2 square periodic basic pattern with no dominant band. Our goal was to stay general and to apply the evaluation on a well-used pattern. But some other tentatives of designing extended pattern have been proposed in the literature [64], for a better spatial distribution of linear polarization filters. An extensive evaluation of best demosaicking algorithms on different arrangements would be considered in a future work.

We found that the acquisition setup may induces correlation between some polarized channels that could be exploited for demosaicking. Since these properties are data-dependent, we have chosen to not use them in our study, despite that they are used in few SFA demosaicking methods.

We remarked that some algorithms need more computation time than others, without necessary giving better results. No computational complexity consideration has been reported in this work. We think that there is a lake of information about these aspects in the original articles. Moreover, Matlab or ImageJ can not provide a consistent evaluation of the complexity of the selected algorithms, e.g., for their potential ability to be parallelized in a hardware acceleration for real-time computing.

## 5. Conclusions

By considering the inter-channel correlation, CFA and SFA schemes aim to improve the spatial reconstruction of channels from the information of other channels. Experiments on the only available polarization image database have shown that such methods provides better results in term of PSNR than PFA-oriented methods. More particularly, we proposed to adapt two CFA demosaicking algorithms based on residual interpolation to the PFA case, and showed that they provide better results than classical PFA-oriented methods. Moreover, the SFA PPID method provides the overall best results, and largely reduces visual artifacts in the reconstructed polarization descriptors in comparison with bilinear method.

Correlation study has shown that the spectral band considered in the acquisition of polarization channels has no influence on the correlation between polarization channels. The correlation results from this study could be an input and provide assumptions for the design of new demosaicking algorithms applied on cameras that mix spectral and polarization filters.

All algorithms were tested on a small database of images. As future work, we hope that an extensive database of polarization and spectral images will be available soon in the research community. Thus, further evaluations on more various materials and image statistics would validate more deeply our conclusions. Furthermore, we believe that a real-time pipelined implementation of the PPID method using GPU or FPGA needs to be investigated, that would be a valuable tool for machine vision applications.

## Figures and Tables

**Table:** (a) 0°

	$C^{1}$	$C^{2}$	$C^{3}$	$C^{4}$	$C^{5}$	$C^{6}$
$C^{1}$	1.0000	0.8848	0.9100	0.7618	0.8571	0.8411
$C^{2}$	0.8848	1.0000	0.9561	0.8956	0.8584	0.8495
$C^{3}$	0.9100	0.9561	1.0000	0.9002	0.9454	0.8846
$C^{4}$	0.7618	0.8956	0.9002	1.0000	0.8352	0.7962
$C^{5}$	0.8571	0.8584	0.9454	0.8352	1.0000	0.8927
$C^{6}$	0.8411	0.8495	0.8846	0.7962	0.8927	1.0000
Spa	0.9691	0.9413	0.9590	0.8770	0.9685	0.9644

**Table:** (b) 45°

	$C^{1}$	$C^{2}$	$C^{3}$	$C^{4}$	$C^{5}$	$C^{6}$
$C^{1}$	1.0000	0.9333	0.9127	0.7949	0.8392	0.8181
$C^{2}$	0.9333	1.0000	0.9620	0.8912	0.8679	0.8245
$C^{3}$	0.9127	0.9620	1.0000	0.9150	0.9417	0.8528
$C^{4}$	0.7949	0.8912	0.9150	1.0000	0.8734	0.7812
$C^{5}$	0.8392	0.8679	0.9417	0.8734	1.0000	0.8664
$C^{6}$	0.8181	0.8245	0.8528	0.7812	0.8664	1.0000
Spa	0.9720	0.9443	0.9624	0.8804	0.9719	0.9747

**Table:** (c) 90°

	$C^{1}$	$C^{2}$	$C^{3}$	$C^{4}$	$C^{5}$	$C^{6}$
$C^{1}$	1.0000	0.9490	0.9133	0.8225	0.8327	0.8102
$C^{2}$	0.9490	1.0000	0.9630	0.8960	0.8687	0.8229
$C^{3}$	0.9133	0.9630	1.0000	0.9335	0.9413	0.8481
$C^{4}$	0.8225	0.8960	0.9335	1.0000	0.9044	0.8024
$C^{5}$	0.8327	0.8687	0.9413	0.9044	1.0000	0.8622
$C^{6}$	0.8102	0.8229	0.8481	0.8024	0.8622	1.0000
Spa	0.9752	0.9550	0.9689	0.9059	0.9757	0.9765

**Table:** (d) 135°

	$C^{1}$	$C^{2}$	$C^{3}$	$C^{4}$	$C^{5}$	$C^{6}$
$C^{1}$	1.0000	0.8961	0.9127	0.7788	0.8545	0.8422
$C^{2}$	0.8961	1.0000	0.9573	0.8974	0.8629	0.8473
$C^{3}$	0.9127	0.9573	1.0000	0.9077	0.9464	0.8827
$C^{4}$	0.7788	0.8974	0.9077	1.0000	0.8501	0.8014
$C^{5}$	0.8545	0.8629	0.9464	0.8501	1.0000	0.8936
$C^{6}$	0.8422	0.8473	0.8827	0.8014	0.8936	1.0000
Spa	0.9687	0.9380	0.9575	0.8688	0.9676	0.9667

**Table:** (e) Average

	$C^{1}$	$C^{2}$	$C^{3}$	$C^{4}$	$C^{5}$	$C^{6}$
$C^{1}$	1.0000	0.9158	0.9122	0.7895	0.8459	0.8279
$C^{2}$	0.9158	1.0000	0.9596	0.8950	0.8645	0.8360
$C^{3}$	0.9122	0.9596	1.0000	0.9141	0.9437	0.8670
$C^{4}$	0.7895	0.8950	0.9141	1.0000	0.8658	0.7953
$C^{5}$	0.8459	0.8645	0.9437	0.8658	1.0000	0.8787
$C^{6}$	0.8279	0.8360	0.8670	0.7953	0.8787	1.0000
Spa	0.9712	0.9446	0.9620	0.8830	0.9709	0.9706

**Table:** (a) *C*^1^

	$I^{0}$	$I^{45}$	$I^{90}$	$I^{135}$
$I^{0}$	1.0000	0.9227	0.8980	0.9763
$I^{45}$	0.9227	1.0000	0.9699	0.9250
$I^{90}$	0.8980	0.9699	1.0000	0.9126
$I^{135}$	0.9763	0.9250	0.9126	1.0000
Spa	0.9691	0.9720	0.9752	0.9687

**Table:** (b) *C*^2^

	$I^{0}$	$I^{45}$	$I^{90}$	$I^{135}$
$I^{0}$	1.0000	0.9262	0.8787	0.9372
$I^{45}$	0.9262	1.0000	0.9470	0.8969
$I^{90}$	0.8787	0.9470	1.0000	0.8974
$I^{135}$	0.9372	0.8969	0.8974	1.0000
Spa	0.9413	0.9443	0.9550	0.9380

**Table:** (c) *C*^3^

	$I^{0}$	$I^{45}$	$I^{90}$	$I^{135}$
$I^{0}$	1.0000	0.9077	0.8960	0.9486
$I^{45}$	0.9077	1.0000	0.9486	0.8970
$I^{90}$	0.8960	0.9486	1.0000	0.9024
$I^{135}$	0.9486	0.8970	0.9024	1.0000
Spa	0.9590	0.9624	0.9689	0.9575

**Table:** (d) *C*^4^

	$I^{0}$	$I^{45}$	$I^{90}$	$I^{135}$
$I^{0}$	1.0000	0.8787	0.8444	0.9317
$I^{45}$	0.8787	1.0000	0.9286	0.8816
$I^{90}$	0.8444	0.9286	1.0000	0.8688
$I^{135}$	0.9317	0.8816	0.8688	1.0000
Spa	0.8770	0.8804	0.9059	0.8688

**Table:** (e) *C*^5^

	$I^{0}$	$I^{45}$	$I^{90}$	$I^{135}$
$I^{0}$	1.0000	0.9074	0.8920	0.9444
$I^{45}$	0.9074	1.0000	0.9524	0.8986
$I^{90}$	0.8920	0.9524	1.0000	0.8955
$I^{135}$	0.9444	0.8986	0.8955	1.0000
Spa	0.9685	0.9719	0.9757	0.9676

**Table:** (f) *C*^6^

	$I^{0}$	$I^{45}$	$I^{90}$	$I^{135}$
$I^{0}$	1.0000	0.9049	0.8155	0.9107
$I^{45}$	0.9049	1.0000	0.8965	0.8823
$I^{90}$	0.8155	0.8965	1.0000	0.8674
$I^{135}$	0.9107	0.8823	0.8674	1.0000
Spa	0.9644	0.9747	0.9765	0.9667

**Table:** (g) Average

	$I^{0}$	$I^{45}$	$I^{90}$	$I^{135}$
$I^{0}$	1.0000	0.9079	0.8708	0.9415
$I^{45}$	0.9079	1.0000	0.9405	0.8969
$I^{90}$	0.8708	0.9405	1.0000	0.8907
$I^{135}$	0.9415	0.8969	0.8907	1.0000
Spa	0.9465	0.9509	0.9595	0.9445

**Table 3:** Summary of the color, spectral, and polarization filter array (CFA/SFA/PFA) interpolation methods. R, A and P abbreviations mean that the algorithms were Re-coded, Adapted, or Provided by the authors of the original work.

Method	Abbr.	Year	Code
**PFA-oriented**			
Bilinear with 5 different kernels [34]	$B_{1-5}$	2009	R
Linear system [35]	LS	2009	R
Adaptive [36]	A	2011	R
Cubic [37]	CB	2011	R
Cubic-Spline [37]	CBSP	2011	R
Intensity Correlation among Polarization Channels [38]	ICPC	2016	P
**CFA-oriented**			
Residual Interpolation [39]	RI	2013	A
Adaptive Residual Interpolation [40]	ARI	2015	A
**SFA-oriented**			
Binary-Three Edge Sensing [41]	BTES	2006	R
Spectral Difference [42]	SD	2006	R
Vector median [43]	VM	2013	P
Discrete Wavelet Transform [44]	DWT	2013	P
Multi Local Directional Interpolation [45]	MLDI	2015	R
Pseudo-Panchromatic Image Difference [11]	PPID	2017	A
Pseudo-Panchromatic Image based Discrete Wavelet Transform [11]	PPIDWT	2017	A

**Table 4:** Demosaicking peak signal-to-noise ratio (PSNR) results, which are averaged over all scenes of the testing database. The descriptors are computed using Equations (1)–(3). The best result for each channel or descriptor is highlighted as bold.

	$I^{0}$	$I^{45}$	$I^{90}$	$I^{135}$	$S_{0}$	$S_{1}$	$S_{2}$	*DOLP*	*AOLP*
**PFA-oriented**									
$B_{1}$	34.88	36.74	36.88	35.87	38.59	36.86	34.99	24.63	17.15
$B_{2}$	37.18	39.44	39.54	38.56	40.77	39.53	37.96	27.11	18.35
$B_{3}$	40.81	44.81	44.99	43.97	44.82	43.87	43.58	31.72	20.66
$B_{4}$	36.87	39.08	39.25	38.19	38.51	43.65	43.80	31.41	20.88
$B_{5}$	36.36	38.40	38.56	37.51	38.27	41.62	40.67	29.51	19.45
LS	x	x	x	x	42.10	41.66	40.20	25.74	18.03
A	40.58	44.75	44.87	43.60	44.61	43.61	43.25	31.69	20.67
CB	41.57	46.59	46.73	45.97	45.94	44.73	45.24	32.66	21.28
CBSP	41.64	47.04	47.15	46.58	46.12	44.82	45.69	32.57	21.28
ICPC	40.98	45.99	46.23	44.95	45.20	44.31	44.68	32.21	20.95
**CFA-oriented (Adapted)**									
RI	42.16	47.06	47.56	47.11	46.17	45.81	**46.94**	**33.77**	21.69
ARI	41.77	46.93	47.39	46.93	45.78	45.52	46.74	33.51	21.60
**SFA-oriented**									
BTES	40.85	45.46	45.82	44.30	44.65	44.60	44.88	32.67	21.33
SD	42.20	43.33	42.81	45.58	45.19	44.14	44.18	26.86	20.69
VM	37.81	39.85	40.05	39.08	40.85	40.70	39.22	28.41	18.77
DWT	40.25	41.02	40.57	41.79	43.08	42.09	41.62	19.85	20.34
MLDI	42.23	44.54	43.93	45.27	45.75	44.53	45.17	30.89	21.50
PPID	**42.60**	**48.34**	**48.00**	**47.66**	**47.02**	**45.85**	46.92	33.16	**22.02**
PPIDWT	40.62	44.60	43.77	43.45	44.97	43.01	42.61	28.72	20.65
